# Buffalo Dental Association

**Published:** 1865

**Authors:** Geo. B. Snow


### BUFFALO DENTAL ASSOCIATION.

Minutes of the meeting held April 3, 1864.

The meeting was called to order by the President, Dr. Geo. E. Hayes.

An essay was read upon "vulcanite work," that being the subject appointed for discussion.

Dr. Hayes asked the opinion of those present, as to the cause of and remedy for the springing of rubber plates. He had been quite interested in Dr. Brown's experiments in the matter, and was sorry the Dr. was not present. His remedy had been to tie the plate upon a second model, obtained from the same impression, and to subject it to a thorough boiling. He very seldom found a vulcanite plate with block teeth that would fit the mouth without some alteration.

Dr. Southwick said he had tried Dr. Brown's plan, which consisted in fastening a stay of stout wire across the wax plate before it was put into the flask, leaving wax enough at the ends of the wire to insure its being easily cut away. The wire was put across between the second molars. After the plate was vulcanized it would perfectly fit the plaster model that it was vulcanized on, but when it was scraped, and the stay was cut out, the heels of the plate would spring together. This would increase the curvature of the palatal portion, and the plate would rock; he then replaced the stay and forced in with it a piece of thick plate, about No. 24 or 25, and heated the set. When it cooled down it would fit the model without the stay.

Dr. Whitney made a few remarks about the early history of rubber in Buffalo. He considers it a great blessing, both to the profession and the patients, and called attention to the state of things which would have existed during the last three years if hard rubber had never been invented. He knew that it had been characterized as a curse to the profession, but he did not agree with this view. He thought dentists could accomplish much more good with it than they could without it. With regard to the springing of the plates, he thought it oftentimes proceeded from opening the flask when it was warm. He thought caution should be exercised in this respect.

Dr. Freeman had often noticed that the molar blocks would crack, without being displaced or apparently injured; would like to know the reason.

Dr. Snow thought it proceeded from putting too much pressure on the rubber. He had been troubled with the blocks cracking, but it was when he had no rule for ascertaining the exact quantity of rubber necessary. He uses a base plate made entirely of wax. When he packs the flask, he weighs the wax taken out, and puts in twice the quantity of rubber. In answer to a question, said he cut large passages for the escape of surplus rubber, cuts a large groove entirely around the flask to within an eighth of an inch of the mould, then cuts runners about a sixteenth of an inch wide, a half an inch apart, from the mould to the groove. He expects to have a little surplus rubber, but it escapes easily. He grinds the joints as close as possible and uses liquid silex in them; has clean joints with very few exceptions indeed.

Dr. Lewis said he used gutta percha plates, and arrived at the same result as Dr. Snow, by weighing and adding half for the rubber. If his gutta percha plate weighed two pennyweights, it would take three pennyweights of rubber to fill the mould.

Dr. Hayes said he used plaster in the joints, grinding it as fine as he could, in a mortar, and mixing it very thin. He applied it to the ends of the blocks as they were put in place for the last time, so that the plaster would not be broken after it set.

On motion:

"The preparation of the mouth for artificial dentures," was made the subject for the next meeting.

The meeting then adjourned.

Geo. B. Snow, *Secretary.*

